# Canine multiple primary tumours: Mammary tubular carcinoma, uterine leiomyosarcoma, and facial sebaceous epithelioma

**DOI:** 10.17221/103/2023-VETMED

**Published:** 2024-03-26

**Authors:** Seung-Hyun Kim, Yeong-Bin Baek, Sang-Ik Park

**Affiliations:** ^1^Laboratory of Veterinary Surgery, College of Veterinary Medicine, Chonnam National University, Gwangju, Republic of Korea; ^2^Laboratory of Veterinary Pathology, College of Veterinary Medicine, Chonnam National University, Gwangju, Republic of Korea; ^3^Laboratory of Veterinary Pathology, College of Veterinary Medicine, Chonnam National University, Gwangju, Republic of Korea; College of Veterinary Medicine and BK21 FOUR Program, Chonnam National University, Gwangju, Republic of Korea

**Keywords:** leiomyosarcoma, mammary gland tumour, multiple origins, primary tumours, sebaceous epithelioma

## Abstract

Multiple primary malignant tumours (MPMTs) are multiple neoplasms with independent pathogenetic origins, placing great importance on the tumorigenesis and clinical treatment. However, due to the rare occurrence and diagnostic confusion, MPMTs have rarely been investigated in veterinary medicine. In this report, a 10-year-old intact female Maltese dog had MPMTs, consisting of two malignant tumours and one benign tumour each derived from a topographically different site: tubular carcinoma in the mammary glands, leiomyosarcoma in the uterus and sebaceous epithelioma in the cheek. The unique combination of MPMTs would be the first case in veterinary research to give insight into the diagnosis, disease characteristics, and surgical treatment.

Recently, in parallel with advances in cancer treatments and prolonged survival, the number of multiple primary malignant tumours (MPMTs) has gradually increased in human and veterinary medicine (Lv et al. 2017). MPMTs are defined by the presence of two or more distinct malignant tumours that have different origins, satisfying three criteria: (1) histopathological confirmation of malignancy of each tumour; (2) topographically separation and difference of each tumour; (3) no metastatic lesion found (Zhai et al. 2018). MPMTs are crucial in investigating the tumour pathogenesis, diagnostic procedures, and clinical treatment. However, due to the rare occurrence and lack of research, MPMTs have rarely been studied in veterinary medicine.

Canine mammary gland tumour (MGT) is the most commonly diagnosed neoplasm, comprising the major proportion of reproductive tumours in female dogs (Kuppusamy et al. 2019). In addition, many retrospective studies have found that MGT has a high malignancy rate (approximately 50%) and a high metastatic potential (almost 50%)., mainly giving rise to mortality in dogs (Im et al. 2014; Feliciano et al. 2023). Therefore, the early diagnosis and surgical resection are imperative, which allow the prevention of tumour progression and better prognosis.

Uterine tumours occur very rarely, less than 0.5% of all canine tumours, usually affecting middle-aged to older female dogs that have not been spayed (Percival et al. 2018). Leiomyoma is the most common, accounting for 90%, while leiomyosarcoma is extremely rare, which grows slowly and possibly invades inside the abdomen (Serin et al. 2010). In some unknown manner, both tumours are endocrine dependent and frequently associated with gynaecological diseases such as ovarian follicular cysts, oestrogen-secreting tumours, endometrial hyperplasia, mammary hyperplasia, and mammary neoplasia (Pena et al. 2006; Tsioli et al. 2011). Mainly, leiomyosarcoma can induce abdominal distension, dyspnoea, and general dullness due to the huge volume occupancy in the abdomen.

Sebaceous epitheliomas are benign tumours and are occasionally found, accounting for 2% of all skin tumours in dogs, which occur most often on the head, ears, and dorsum between 8 and 13 years of age (Goldschmidt and Hendrick 2002; Giuliano et al. 2009). The histopathological findings are characterised by moderate lobular irregularity, and basaloid cell proliferation with few well-differentiated sebocytes surrounded by interlobular stroma (Yoon and Par 2016).

The patient received surgical treatments on three tumours having either cancerous or non-cancerous lesions depending on the mammary gland, uterus, and cheek.

The histopathological examination and morphological classification confirmed a case of MPMTs composed of two concurrent primary malignant tumours with sebaceous epithelioma.

## Case presentation

A 10-year-old intact female Maltese dog was presented with multiple masses in the 4^th^ and 5^th^ mammary glands and the left cheek. The masses in the mammary gland units were firm and mobile without any significant signs in the axillary and inguinal lymph nodes on both sides by physical examination. On the other hand, the cheek mass was soft, mobile, and measured at around 4–5 cm in diameter, causing severe pruritus and ulceration.

**Figure 1:**
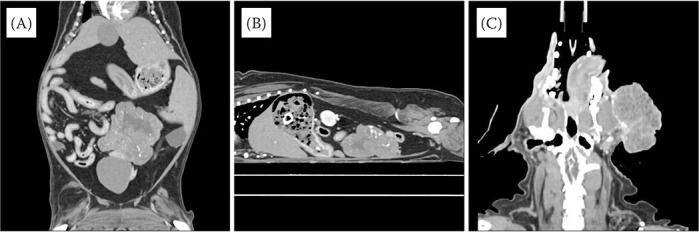
CT scan of the abdomen and the skull (A,B) The abdominal mass (6.7 × 6.8 × 3.8 cm) was multilobulated and located between the splenic tail and urinary bladder. (C) The facial mass (3.9 × 3.8 × 5.3 cm) has an irregular margin at the lateral end of the left zygomatic arch, showing a clear border with the adjacent masseter muscle

Laboratory tests revealed that the biochemistry and electrolytes were normal. However, the complete blood count (CBC) detected non-regenerative anaemia and severe leucocytosis along with moderate neutrophilia. Moreover, the radiography, ultrasonography, and computed tomography (CT) scans found masses in the abdomen and cheek (Figure 1). The abdominal mass was measured at around 7 cm in diameter and multilobulated between the splenic tail and urinary bladder, while the facial mass was around 4 cm with an irregular margin. In this case, a partial mastectomy and facial tumorectomy, along with an exploratory laparotomy and ovariohysterectomy, were considered the best therapeutic option. After the surgical treatment, each collected mass was subject to a histopathological examination.

**Figure 2:**
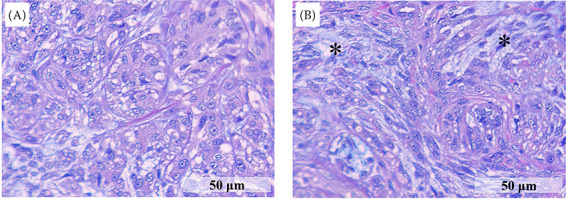
The histopathological features of the MGT diagnosed as tubular carcinoma with grade II malignancy (A) Tumour lesions mainly consist of neoplastic epithelial cells with minimal tubular differentiation. (B) Occasional proliferation of myoepithelial cells showing a mild mucinous change (asterisk)

The mammary gland lesions were mainly composed of poorly differentiated tubular structures (Figure 2). There was minimal tubular differentiation, but occasional lumens were present. Individual neoplastic cells showed moderate anisocytosis and anisokaryosis and had oval-shaped vesicular to chromatic nuclei and a fairly extensive amount of cytoplasm. The MGT was diagnosed as tubular carcinoma with grade II malignancy according to the staging system for canine MGT (Misdorp et al. 1999).

**Figure 3:**
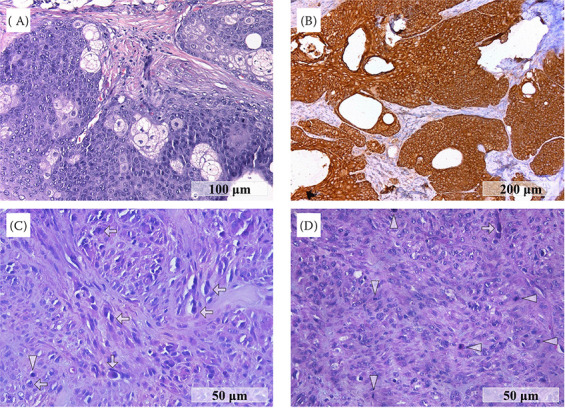
Tumour lesions in the sebaceous gland (A,B) and uterus (C,D) (A) Tumour lesions are composed of basophilic reserve cells with occasional sebaceous differentiation. (B) Most neoplastic cells were found to be positive for pan-cytokeratin (AE1/AE3) by the IHC. (C) Highly pleomorphic neoplastic cells in a fascicular pattern often show bizarre tumour cells with very big nuclei (arrow). (D) Numerous mitoses (arrowhead) were found throughout the lesions

The facial tumour lesions were mainly composed of basaloid reserve cells in islands or trabeculae, which were polyhedral shaped and had scant to moderate amphophilic cytoplasm (Figure 3). The nuclei were large and vesicular and had multiple small nucleoli. There were scattered small foci of sebaceous differentiation, characterised by clusters of cells with lipidised cytoplasm, indicating morphological features of a benign tumour. The immunohistochemistry (IHC) confirmed that the majority of the neoplastic cells were positive for pan-cytokeratin, leading to a diagnosis of sebaceous epithelioma.

In the uterus, the endometrium had cystic endometrial hyperplasia, resulting in moderate mucometra and endometritis. The tumour lesions mainly comprised of spindle-shaped or polygonal cells with abundant, wispy cytoplasm and indistinct cell borders with a high cellular density (Figure 3). Nuclei highly varied in size and shape, some of which were cigar to spindle-shaped, and others vesicular with indistinct nucleoli. Occasionally bizarre tumour cells with big nuclei were found throughout the lesions. Furthermore, numerous mitoses were observed, including atypical figures. The IHC against desmin confirmed a diagnosis of leiomyosarcoma (data not shown).

## DISCUSSION

MPMTs are defined by the presence of two or more distinct malignant tumours that have a pathogenetic origin in multiple organs, which could be synchronous or metachronous, occurring within 6 months from the diagnosis of a previous malignant tumour or 6 months after the first diagnosed tumour, respectively (Zhao et al. 2015; De Luca et al. 2019). Previously, the histopathological examination confirmed two malignant tumours in topographically separated two organs: the tubular carcinoma of the mammary gland and uterine leiomyosarcoma. The tumours were not related to each other, leading to a diagnosis of MPMTs along with the sebaceous epithelioma of the cheek. However, we could not define its tumorigenesis due to a lack of information about the outpatient.

In clinical oncology, the most crucial part of applying appropriate treatment is classifying the tumour’s origin site and defining its stage and grade correctly. The difficulties in distinguishing between MPMTs and metastatic secondary tumours provide challenges to clinicians. Without the identification of the encountered tumours, the optimal treatment cannot be achieved. In human medicine, MPMTs have been studied thoroughly, while there are many limitations in veterinary medicine despite the clinical importance of MPMTs. The incidence of MPMTs was between 0.734% and 11.7% in 1 104 269 humans (Demandante et al. 2003), and was 3% in 1 722 dogs (Rebhun and Thamm 2010).

Fortunately, an exploratory laparotomy helped to acquire the uterine neoplasm, resulting in the successful tumorectomies of three tumors originating from three different sites. In the present case, no metastasis was found in the MGTs, but the uterine leiomyosarcoma was highly suggestive of metastasising due to multiple enlarged lymph nodes according to an abdominal CT scan. To the best of our knowledge, this is the first case of a unique combination of three independent tumours in veterinary medicine, including the mammary gland, uterus, and facial sebaceous gland. Still, we have several limitations: (1) the lymphadenectomy of the metastatic lymph nodes was not available due to poor accessibility and increased burden to the geriatric patient, leaving the exact metastatic lesion of the lymph nodes undefined; (2) the origin of abdominal incidentaloma shown by the diagnostic imaging did not correspond to the actual tumour origin site; (3) although new strategies treating mammary cancer have been developed (Vazquez et al. 2023), we could not apply any immunotherapy or targeted therapy against the specific protein due to the owner’s unwillingness.

Nonetheless, an optimal clinical treatment was designed and performed. The patient has been doing well without any signs of recurrence or distant metastasis for over two years, confirmed by periodic physical examinations, radiography, and sonography. Furthermore, it is a very encouraging result considering that the average 1-year survival rate of patients with mammary cancer is 19% and that of patients with leiomyosarcoma is 75%, respectively (Rasotto et al. 2017; Cohen et al. 2003). It shows the surgical resection of each neoplasm to be clinically significant when treating concurrent tumours.

Additionally, the medical imaging alone would not be sufficient enough to identify a tumour’s origin and distinguish between primary and metastatic tumours. Any further studies on MPMTs should include research for the biological, clinical, pathological, and molecular characteristics, providing optimal diagnostic and therapeutic options as studied in mammary cancer (Vazquez et al. 2023). In this context, we believe the present case would contribute to establishing a standardised diagnosis and medical procedures for MPMTs, appealing to clinicians and researchers in veterinary medicine.
